# Schizophrenia pathophysiology: are we any closer to a complete model?

**DOI:** 10.1186/1744-859X-8-12

**Published:** 2009-05-15

**Authors:** Shaheen E Lakhan, Karen F Vieira

**Affiliations:** 1 Global Neuroscience Initiative Foundation, Los Angeles, California, USA

## Abstract

Schizophrenia, a severe brain disorder that involves hallucinations, disordered thinking and deficiencies in cognition, has been studied for decades in order to determine the early events that lead to this neurological disorder. In this review, we interpret the developmental and genetic models that have been proposed and treatment options associated with these models.

Schizophrenia was initially thought to be hereditary based on studies of high incidence in certain families. Additionally, studies on specific genes such as *ZDHHC8 *and *DTNBP1 *seem to suggest susceptibility to the onset of this disorder. However, no single gene variation has been linked to schizophrenia, and recent evidence on sporadic cases of schizophrenia refutes genetics as being a singular cause of the disease. In addition, current data suggests neurodevelopmental or environmental causes such as viral infections and prenatal/perinatal complications.

Before any brain disorder can be understood, however, multiple cognitive neuroscientific models that accommodate evidence from many biomedical research fields should be considered, and unfortunately, many of these models are in the earliest stages of development. Consequently, it makes us question whether we are any closer to an adequate understanding of the pathophysiology of schizophrenia.

## Background

Schizophrenia is the term used to describe a mental disease which has a spectrum of symptoms, including alterations in perception, thought and sense of self, decrease in volition, psychomotor slowing, and displays of antisocial behavior [1]. Schizophrenia is a heterogeneous disease, making it difficult for clinicians to pinpoint the precise neuropathology underlying its extensive array of symptoms. It has been well accepted that schizophrenia can result from single or multiple disorders within discrete regions of the brain. A number of models have been proposed to explain the mechanism for the development of schizophrenia in terms of the nature, timing and the course of brain changes; processes which are still not well understood. In this review, the major models for the cause of schizophrenia are summarized, as well as the potential links between brain structures and neuronal signaling and the development of schizophrenia. In order to improve treatment options and prognostic outcomes for schizophrenia it is necessary to understand the pathophysiology that contributes to this disease state.

## Neurodevelopmental hypothesis

Based on early studies, it was believed that the structural brain changes that occur in schizophrenia were caused by early prenatal or perinatal insults, which can present a predisposition to the development of schizophrenia. Complications in pregnancy can alter the organization of the axonal connection patterning in synaptic projections by affecting neuronal cell proliferation, migration and apoptosis, processes which are equally required for proper central nervous system (CNS) development. As early as 1976, it was reported that cerebral ventricles or cortical sulci are enlarged in many schizophrenia patients even during early stages of the disease [2]. Studies in the late 1980s by Weinberger, as well as Murray and Lewis, proposed that the predisposition to schizophrenia is highly dependent on defects in early brain development, which can lead to specific patterns of brain dysfunction [3,4]. Weinberger's findings suggest that schizophrenia occurs from non-specific histopathology that exists in the limbic system, diencephalon, and prefrontal cortex of the brain. The pathology occurs so early in development that the actual injury occurs long before the diagnosis is made. He also reported that later in life, those injuries or lesions interact with normal brain maturational events, particularly within the dorsal prefrontal cortex and dopaminergic neural systems [4]. Much of the focus of early studies examined defects in the left cerebral hemisphere in schizophrenia. However, evidence also supports an increased likelihood that schizophrenic patients are left-handed [3], as there exists a gene *LRRTM1 *associated with left-handedness and which promotes brain asymmetry, a noted characteristic among many schizophrenic patients.

Similar to Weinberger's theory on susceptibility to schizophrenia, Benes *et al*. examined the anterior cingulate cortex (ACC) of postmortem schizophrenic brains. This study suggested that the development of schizophrenia was related to congenital abnormalities involving reduced number and altered interconnectivity of neurons in the ACC [5]. Benes *et al*. also speculated that such abnormalities give rise to schizophrenia-like symptoms during late adolescence and early adulthood, because this is the period of increased myelination of the perforant pathway [6]. This pathway carries fibers from the entorhinal cortex to the hippocampus and when activated, may trigger the expression of abnormalities in the cortical regions as they interrupt corticolimbic circuitry [5]. Similarly, McGlashan and Hoffman also suggested a model of schizophrenia that involved this early prenatal-neurodevelopmental insult. However, this study described schizophrenia as a disorder of developmentally reduced synaptic connectivity that arises from developmental disturbances of synaptogenesis during the prenatal period and/or synaptic formation during adolescence [7].

More recently, Pantellis *et al*. have provided evidence to support the neurodevelopmental hypothesis for schizophrenia. Their studies suggested that schizophrenia is a disease resulting from limited progressive brain changes that occur during prenatal development and in stages prior to the onset of psychosis [8]. Their research indicated that schizophrenic brains lacked the 'normal' leftward ACC sulcal asymmetry, a result of reduced folding in the left ACC. The sulcal/gyral folding is almost complete by the third trimester of gestation and is relatively stable after birth. They suggested that it is abnormal ACC folding that contributes to the etiology of schizophrenia [1].

## Contributing environmental factors

Epidemiologic studies, as well as studies from discordant identical twins, indicate that there are significant environmental risks for schizophrenia which exert pronounced effects on early brain development. Prenatal exposure to viral infections such as influenza and poliovirus, poor prenatal nutrition, adverse obstetric events and cannabis smoking during adolescence, are all examples of environmental factors, which may increase the risk of schizophrenia. It has been suggested that environmental factors combined with a genetic predisposition result in the manifestation of schizophrenia [3].

## Impairments in cognitive function

Schizophrenia is marked by severe cognitive dysfunction or impairment. Specifically, individuals with schizophrenia are unable to think clearly, have problems with memory, critical thinking and problem solving, are unable to quickly process information, and have dysfunction in the ability to initiate speech. Older models for the development of schizophrenia suggest that brain lesions result in structural abnormalities, which eventually lead to these cognitive deficits.

Currently, investigators are using functional imaging techniques to help improve the understanding of schizophrenia. Functional magnetic resonance imaging (fMRI), combined with other diagnostic tools such as the electroencephalogram (EEG) have allowed for the precise examination of major psychiatric illnesses. Functional neural imaging, particularly fMRI, has proven to be a powerful tool to gain understanding of schizophrenia as this technique allows for high spatial and temporal resolution in studies examining cognitive dysfunction and mapping of patient brains [9,10]. Using functional imaging, Honey and Fletcher hypothesized that the biological basis of schizophrenia includes disruptions in working memory and reduced working memory capacity [11]. A study by Keefe *et al*. showed that cognitive dysfunction is highly correlated with the incidence of schizophrenia. This study found that 98.1% of people with schizophrenia performed below expected on cognition based on predictions from Wide Range Achievement Test – Revision 3 (WRAT-3) reading tests or parental education [12]. Based on this schizophrenic model, pharmacological, as well as non-pharmacological treatment methods such as education and neurocognitive activation have been used to improve cognitive reserve or function [13].

Wolf *et al*. suggested that the working memory deficit is at the core of the cognitive impairment in schizophrenia, and that this lead to the higher deficits observed in schizophrenia patients. fMRI was used to associate working memory deficits with problems of the prefrontal cortex [14]. In addition to the prefrontal cortex, other investigators concluded that the superior temporal areas and the striatum were also highly involved in the dysfunction of working memory. In these studies, schizophrenic patients meeting Diagnostic and Statistical Manual of Mental Disorders 4th Edition (DSM-IV) criteria for schizophrenia showed less activation in frontoparietal and subcortical regions compared to healthy subjects. Compared to patients with depression, schizophrenia patients also had less prefrontal activation in the left inferior frontal cortex and right cerebellum as well as a lack of deactivation of the superior temporal cortex [15].

A study by Barch and Csernansky found that there was a similar level of activation in several regions of the brain for working memory tests, both verbal and non-verbal, in healthy subjects as to what is seen in schizophrenic patients, but that healthy individuals have an increased activation in the parietal and left ventral prefrontal cortex when testing verbal working memory. This study also showed that individuals with schizophrenia have bilateral defects in dorsal frontal and parietal activation during both verbal and non-verbal working memory tasks [16]. These investigators also demonstrated that patients with schizophrenia have greater activity for verbal working memory in the ventral prefrontal and parietal regions, than for non-verbal working memory. However, these individuals showed less verbal superiority in a left ventral prefrontal region. This led the researchers to conclude that working memory deficits in individuals with schizophrenia reflect mostly the inability to activate areas of the brain that are associated with the central executive components of working memory rather than domain-specific storage buffers [16].

## Oligodendrocytic computation capacity theory

White matter abnormalities in the brain have also been correlated with schizophrenia. The net result of these abnormalities is specific defects in brain lateralization. Some investigators have suggested that damaged or immature oligodendrocytes can prevent or hamper the properties of axonic formation. Based on this, Mitteraue postulated the oligodendrocytic computation capacity theory, which ascertains that decomposition of the oligodendrocyte-axonic system may be responsible for symptoms leading to complete incoherence as seen in schizophrenia [17]. This is also extended to astrocyte-neuronal interactions in tripartite synapses. In line with this argument, Mitterauer stated that all macroglial cells with their syncytia must be considered in their interactions with the neuronal system [17].

## Genetic inheritance in schizophrenia

Schizophrenia manifestations are more common in some families. Although not strictly due to heredity, newer models have been proposed that suggest that specific allelic inheritance may contribute to the development of schizophrenia. Recent studies of twins and adoption studies support that schizophrenia is, at least partially, a genetic disorder [18]. Foley *et al*. suggest that schizophrenia may be a complex, multigene trait. The alleles are present in the population and, when expressed individually, may have a relatively weak effect; however, they can interact synergistically when expressed together. From this observation, it has been theorized that there is incomplete penetrance of the full disorder, or inherited alleles are often insufficient in number, but the individual still manifests the classical clinical symptoms with varying behavioral phenotypes. To date, many vulnerability genes have been identified but none have been conclusively linked to schizophrenia [18].

The current view is that the total susceptibility effect arises from a collection of small individual effects. Based on current evidence, it has been suggested that individuals with schizophrenia have risk genes, which impact their neurodevelopmental mechanisms, and this subsequently results in inefficient or disturbed neuronal communication later in life. Foley *et al*. reason that such neurodevelopmental errors occurring from normal single nucleotide polymorphisms (SNPs) and copy number variants (CNVs) within the population, or mutations such as insertions/deletions can alter single or multiple metabolic or cellular processes. These different mutations may all ultimately lead to manifestation of schizophrenic symptoms [18].

Several major processes have been identified and are implicated as schizophrenia risk genes. The current view is that most of these genes can exert small individual effects and can aggregate by chance, associative mating or other mechanisms constituting increased risk for schizophrenia. Bridging the older models, these genes may be affecting changes in attention, memory, language, or other cognitive functions through small effects on neurotransmitter function, cerebral structural organization, brain metabolism, or connectivity, as they interact with other non-genetic factors.

Foley *et al*. suggested that the inheritance variation and selection of schizophrenia operates more through a Darwinian mechanism rather than a Mendelian mode of inheritance. It has also been hypothesized that schizophrenia has been the psychiatric result of a gene that confers disease risk in the current environment, but that it may have provided a survival and/or reproductive advantage in an evolutionarily ancestral environment [18]. Supporting the theory of inheritance in gene susceptibility, Crow proposed that that the susceptibility genes for schizophrenia were inevitable 'trade-offs' for adaptations related to the development of language by humans [19].

In 2002, Straub *et al*. isolated a suspected schizophrenia susceptibility gene named *DTNBP1 *[20]. This is considered a gene for high schizophrenia susceptibility as determined by systematic linkage disequilibrium mapping across a linkage region on chromosome 6p in 270 affected families from the Irish Study of High Density Schizophrenia Families. The exact gene function, expression and interactions with other molecules in the cell have not been completely elucidated. It has been suggested that there is a reduced expression of *DTNBP1 *in the frontal cortex and hippocampal formation of schizophrenia patients [21]. Additionally, a few non-synonymous amino acid changes have been observed in its gene product, dystrobrevin binding protein 1, in the human population, but none of these have been definitively associated with schizophrenia [22].

In a recent review, Gogos and Gerber described how many other susceptibility genes have been identified in the development of schizophrenia. One of the affected proteins was proline dehydrogenase (PRODH), an enzyme that metabolizes l-proline, a neuromodulatory amino acid that is directly involved in glutamate-mediated transmission. *PRODH *has been frequently found deleted in schizophrenia patients, suggesting it plays a significant role in the pathophysiology of schizophrenia. Family samples from parents to affected children were examined for the specific transmission of 72 SNPs and multi-SNP haplotypes, and investigators identified the transmission of a gene variant located at the 3' end of the *PRODH *gene. This finding was later replicated in two independent family-based samples. Functional analysis has linked several of these variants with pronounced decreases in enzymatic activity. Based on mouse models, PRODH-deficiency showed physiological problems of cortical dopamine turnover and transmission that is similar to schizophrenia in humans [23].

The gene *DAOA*, also known as *G72*, has also been shown to have a significant association with schizophrenia. Both expression and functional studies indicate that the gene product, D-amino acid oxidase activator, may have an important interaction with an amino oxidase to modulate its enzymatic activity. This could be important in glutamate signaling, an important pathway affected in most schizophrenia patients [24].

*TAAR6*, the gene that encodes the trace amine associated receptor 6, was also identified as another susceptibility gene. *TAAR6 *was originally identified in families with schizophrenia. *TAAR6 *is a G-protein-coupled receptor that is widely expressed in the brain [23].

More recently, it was also shown that a deletion in the *ZDHHC8 *gene affects the ratio of an intron-4-containing unspliced form, resulting in the encoding of a truncated inactive form of the transmembrane palmitoyltransferase that modifies postsynaptic density (PSD) proteins such as PSD-95. These enzymes have important roles in excitatory synaptic transmission of the human brain. Subtle changes in the residues of this enzyme have been shown to lead to changes in its activity. This has been shown to cause a 1.5-fold increase in disease risk in two of the families tested. Splice variants of *ZDHHC8 *or changes in its expression level have also been shown to have a significant role in modulating the development of schizophrenia especially in individuals with 22q11 deletions [25].

The neureguline1 gene (*NRG1*) is one of the most commonly evaluated genes in schizophrenia research. Previous studies suggested that one SNP of this gene could be involved in the development of schizophrenia. However, studies published in 2009, suggest that multiple SNPs of the *NRG1 *gene might cause schizophrenia in certain groups of people, but that population stratification also plays a role in the onset of schizophrenia [26]. Recent studies also suggest that *NRG1 *SNPs cause speech impairments on a semantic level. More specifically, as the number of harmful alleles increases, verbal performance decreases. Such findings might begin to explain the various cognitive difficulties that are caused by schizophrenia [27].

Catechol-*O*-methyltransferase (COMT) is an enzyme that plays a role in catecholamine metabolism in the brain. Studies have shown that schizophrenia patients have increased levels of the *COMT *gene in glial cells located in the frontal cortex. Increased *COMT *expression in schizophrenia patients may be responsible for disrupted dopamine-glutamate interactions and glial abnormalities [28]. *COMT *polymorphisms also appear to disturb neurocognitive functions and by doing so increase susceptibility to schizophrenia [29].

Disrupted in schizophrenia 1 (DISC1) is a protein with a wide array of functions suspected to be involved in the pathogenesis of schizophrenia. Decreased levels of the *DISC1 *gene in the brain cause abnormal growth, disrupted migration, and accelerated integration of adult neurons. Abnormalities such as these can lead to seizures and may be involved in the development of schizophrenia [30,31].

## Reduction in neuropeptide Y

Several studies have shown a clear relationship between reduced levels of neuropeptide Y (NPY) in the brain and the pathophysiology of schizophrenia. Two independent groups have reported a reduced NPY content in the postmortem brains of schizophrenics [32,33]. Yet another research group had reported that the NPY mRNA levels in the frontal cortices of schizophrenics were significantly reduced compared with those of matched controls [34].

Studies undertaken by Itokawa *et al*. showed that a decreased amount of NPY in the brain of schizophrenics is a pathogenic change and that the *NPY *gene may be a susceptibility gene for schizophrenia. This group was the first to find polymorphisms in four loci in intron 1 and two loci in the promoter region of NPY corresponding to a change in genotype at -485C>T. These results suggest that the decreased NPY level as seen in the postmortem brain is probably genetically determined in specific subsets of schizophrenics [35].

## Alterations in neurotransmission

There has been extensive evidence that glutamatergic *N*-methyl-D-aspartate (NMDA) neurotransmission is also highly disrupted in schizophrenia. Spinophilin, a neuronal protein implicated in the regulation of NMDA signaling, was also reported to be downregulated in the striatum after repeated phencyclidine (PCP) treatment. These results demonstrated that repeated treatment PCP drugs, an NMDA receptor antagonist, could produce specific cognitive deficits that are associated with alterations in gene expression in brain regions that appear to play a significant role in the pathophysiology of schizophrenia [36].

Other studies indicated that dopamine D2 receptor expression is also highly implicated in the disturbance associated with schizophrenia. In studies using transient overexpression of D2 receptors in the striatum of transgenic mice, abnormal prefrontal cortex function was observed. Supporting this finding, studies in primary neurons showed that the siRNA knock-down of dysbindin, a protein thought to modulate D2 but not D1 receptor internalization and signaling, resulted in reduced glutamate release. This suggests that decreased dysbindin may decrease exocytosis of glutamate-containing synaptic vesicles, which alter neuronal transmission and may be responsible for the disturbances associated with schizophrenia. *In vitro *studies using the rat pheochromocytoma P12 cell line siRNA to dysbindin was also shown increase dopamine secretion. *In vivo*, dopaminergic transmission and turnover is increased in the cortex of the dysbindin mutant mice with decreased dopamine levels [37].

Gamma-aminobutyric acid (GABA) has also been associated with the development of schizophrenia. Schizophrenia patients exhibit expression insufficiencies in GABA transcripts that encode GABA neurons, certain GABA(A) receptor subunits and regulators that are involved in GABA neurotransmission. Such abnormalities cause cognitive function impairments that typically affect working memory in schizophrenia patients. To date, several studies suggest that altered GABA neurotransmission, particularly in the dorsolateral prefrontal cortex, leads to impaired working memory in patients with schizophrenia [38].

## Involvement of phosphatidylinositol signaling

In more recent studies, phosphatidylinositol-4-phosphate 5-kinase (PI4,5K) has been strongly associated in the incidence of schizophrenia and its involvement has been replicated in several studies. The activation of KCNQ, a potassium ion channel regulated by phosphatidylinositol signaling, can weaken the central stimulating effects of the neurotransmitter dopamine, and stimulant drugs such as cocaine, methylphenidate, and PCP. In one study, investigators were able to explore the functional relevance of *PIP5K2A*, the gene encoding PI4,5K. In this study, the effects of the neuronal *PIP5K2A *on a combination of KCNQ subunits (KCNQ2, KCNQ5, KCNQ2/KCNQ3, and KCNQ3/KCNQ5) in a *Xenopus *expression system were closely examined. They found that wild type PIP5K2A, but not the schizophrenia-associated mutant (N251S)-*PIP5K2A*, was able to activate the heteromeric KCNQ2/KCNQ3 and KCNQ3/KCNQ5 channel complexes that make up the neuronal voltage-gated potassium M channels. Homomeric KCNQ2 and KCNQ5 channels were not activated by this kinase, suggesting that the KCNQ3 subunit is important for *PIP5K2A*-mediated effects. From the acute application of PI(4,5)P2 and a PIP2 scavenger they conclude that the mutation N251S in schizophrenia renders the kinase *PIP5K2A *inactive. These results suggested that the schizophrenia-linked mutation of the kinase results in reduced KCNQ channel function and this could explain the loss of dopaminergic control [39].

## Drugs used to treat schizophrenia

Older antipsychotic medications including chlorpromazine, haloperidol, perphenazine and fluphenazine are known to cause extrapyramidal side effects, such as rigidity, persistent muscle spasms, tremors, and restlessness. During the 1990s, newer atypical antipsychotics with very little to no side effects were developed. The first of this new class of antipsychotic drugs was clozapine (CLZ), which reduced the motor side effects, cognitive deficits and even suicidal tendencies associated with anti-schizophrenic drugs. The use of CLZ has especially been clinically effective in patients who experienced past treatment resistance. The active CLZ metabolite *N*-desmethylclozapine (NDMC) may play a role in mediating the efficacy of CLZ since it is metabolically active and capable of binding the sites of its parent compound [40]. After clozapine, additional drugs including risperidone, olanzapine, quetjapine, and ziprasidone were used to treat schizophrenic patients.

As previously stated, NMDA receptors are believed to also be involved in the long-term potentiation and memory consolidation processes in humans and this pathway has been reported to be deregulated in models of schizophrenia. Phosphodiesterase 5 (PDE5) inhibitors have been shown to increase the cyclic guanosine monophosphate (cGMP) concentrations, and thus signaling, in the intracellular pathway activated by NMDA receptors.

In particular one PDE5 inhibitor, sildenafil has been shown to enhance memory in various animal models. In 1 study, 17 adult schizophrenia outpatients were treated with a single oral dose of placebo, or sildenafil at 50 mg, and sildenafil at 100 mg after every 48 h. In this study, the psychiatric symptom ratings and a cognitive battery testing were performed first at baseline and then 1 h following drug or placebo administration. Additionally, the memory consolidation was examined by testing recall 48 h later but prior to the next drug administration. However, neither 50 mg nor 100 mg doses of sildenafil significantly affected cognitive performance or symptom ratings when they compared them to the patients that were administered placebo. From these results, the authors concluded that although sildenafil acts as a cognitive-enhancer in animal models, this strategy for treating putative NMDA receptor-mediated memory deficits might not be successful in human models. However, it was possible that the doses they used may not have been optimal or that repeated dosing may be necessary to achieve a therapeutic effect [41].

Another drug, aripiprazole, is an atypical antipsychotic drug shown to improve the disruption of prepulse inhibition and social interaction in various animal model of schizophrenia that have been induced by PCP. In one study, researchers examined the effect of aripiprazole on the cognitive impairment in mice treated with PCP repeatedly. To do this, they repeatedly administered PCP (10 mg/kg for 14 days) to mice followed by an assessment of their cognitive function using a novel-object recognition task. The therapeutic effects of aripiprazole (0.01 to 1.0 mg/kg) and haloperidol (0.3 and 1.0 mg/kg) on cognitive impairment in mice treated with PCP was then assessed. They found that single (1.0 mg/kg) and repeated (0.03 and 0.1 mg/kg, for 7 days) treatment with aripiprazole reduced PCP-induced impairment of recognition memory. In addition both the single and repeated treatment with haloperidol (0.3 and 1.0 mg/kg) failed to decrease PCP-induced cognitive impairment.

To establish the exact mechanism of aripiprazole on recognition memory in PCP-treated mice, they performed cotreatment with a dopamine-1 receptor antagonist, SCH23390, and a serotonin 5-hydroxytryptamine (5-HT)(1A) subtype receptor antagonist, WAY100635. They found that the effect of aripiprazole on recognition memory in PCP-treated mice involved dopamine receptors and serotonin 5-HT(1A) receptor subtypes. It did not involve the D2 receptors since cotreatment with a D3 receptor antagonist, raclopride, did not alter the effect of aripiprazole. These results suggest that the ameliorative effect of aripiprazole on PCP-induced memory impairment is associated with dopamine D1 and serotonin 5-HT(1A) receptors only [42].

## Conclusion

In summary, several models have been presented in research studies to explain the disabling and complex disorder schizophrenia (Table 1). Initial reports indicated that schizophrenia was the result of insults occurring during the early or even late stages of pregnancy, creating histopathological damage to specific areas of the brain. Additionally, exposure to significant environmental factors has been shown to lead to the development of schizophrenia. Apparent enlargement and lack of symmetry of certain brain regions discovered postmortem validate this model. Another model suggests that impairments in cognitive function explain the reduced working memory capacity and severe cognitive dysfunction of schizophrenia. Genetic inheritance is thought to contribute, at least partially, to the development of schizophrenia, and newer models of the disease are identifying susceptibility genes where mutations may increase disease risks by changing enzymatic activity or modulating neuronal signaling. The development of antipsychotics relies on these models of schizophrenia in order to accurately address the pathophysiological properties of the disease. Despite many contradictions in these models, important details involving the neuropathology of the brain give us hints about the events leading up to the disturbances in neurological transmission associated with schizophrenia.

**Table 1:** Pathophysiological models of schizophrenia and their associated drugs

Models	Relevant drugs	References
Development of brain changes due to prenatal or perinatal insult(s); neurodevelopment hypothesis		[1-4,8]
Due to disruption of working memory leading to reduced working memory even with normal brain function		[10-15]
Genetic inheritance contributes to the onset of schizophrenia		[18,19]
Decomposition of the oligodendrocyte-axonic system causes symptoms of schizophrenia; oligodendrocytic computation capacity theory		[17]
The *NPY *gene is a susceptibility gene for schizophrenia; reduction of levels of neuropeptide Y (NPY) in the brain leads to pathological changes		[26,27,29]
Reduced expression of *DTNBP1 *confers susceptibility to schizophrenia		[21,22]
*PRODH *is deleted in schizophrenia patients and may play a significant role in the pathophysiology of schizophrenia.		[23]
*DAOA *(*G72*) produces a gene product that affects glutamate signaling in schizophrenia patients		[24]
*TAAR6 *is a familial gene that is constitutively expressed in the brain and was discovered in families who had a history of schizophrenia		[23]
Expression changes or splice variants of *ZDHHC8 *gene leads to the disruption of excitatory synaptic transmission in the brain and increases the risk of developing schizophrenia		[25]
Multiple single nucleotide polymorphisms (SNPs) of *NRG1 *appear to cause speech deficits on the semantic level and might increase the susceptibility to schizophrenia		[26,27]
Increased levels of *COMT *are associated with glial abnormalities and altered dopamine-glutamate interactions		[28,29]
Decreased levels of *DISC1 *cause neuronal abnormalities that might play a role in schizophrenia pathogenesis		[30,31]
Due to alterations in glutamatergic *N*-methyl-D-aspartate (NMDA) neurotransmission	Phencyclidine (PCP) drugs	[40]
Dopamine D2 receptor expression is also highly implicated in the disturbance associated with schizophrenia	Aripiprazole, haloperidol	[37,42]
Phosphatidylinositol-4-phosphate 5-kinase (PI4,5K) has been strongly associated in the incidence of schizophrenia	Sildenafil	[41]

## Abbreviations

ACC: anterior cingulate cortex; cGMP: cyclic guanosine monophosphate; CLZ: clozapine; COMT: catechol-*O*-methyltransferase; CNV: copy number variants; DAOA: D-amino acid oxidase activator; DISC1: disrupted in schizophrenia 1; DSM-IV: Diagnostic and Statistical Manual of Mental Disorders, 4th edition; DTNBP1: dystrobrevin-binding protein 1; fMRI: functional magnetic resonance imaging; GABA: gamma-aminobutyric acid; KCNQ: potassium channel, voltage-gated, KQT-like subfamily; LRRTM1: leucine-rich repeat transmembrane protein 1; NDMC: *N*-desmethylclozapine; NMDA: glutamatergic *N*-methyl-D-aspartate; NPY: neuropeptide; NRG1: neureguline1; PCP: phencyclidine; PDE5: phosphodiesterase 5; PI4,5K: phosphatidylinositol-4-phosphate 5-kinase; PIP5K2A: phosphatidylinositol-4-phosphate 5-kinase = type II; PRODH: proline dehydrogenase; SNP: single nucleotide polymorphisms; siRNA: small interfering ribonucleic acid; TAAR6: trace amine associated receptor 6; WRAT-3: Wide Range Achievement Test, 3rd edition; ZDHHC8: zinc finger DHHC domain-containing protein 8.

## Competing interests

The authors declare that they have no competing interests.

## Authors' contributions

SL and KV participated in the preparation of the manuscript. Both authors read and approved the final manuscript.
